# Influence of Ecto-Nucleoside Triphosphate Diphosphohydrolase Activity on *Trypanosoma cruzi* Infectivity and Virulence

**DOI:** 10.1371/journal.pntd.0000387

**Published:** 2009-03-03

**Authors:** Ramon F. Santos, Marcela A. S. Pôssa, Matheus S. Bastos, Paulo M. M. Guedes, Márcia R. Almeida, Ricardo DeMarco, Sergio Verjovski-Almeida, Maria T. Bahia, Juliana L. R. Fietto

**Affiliations:** 1 Núcleo de Pesquisa em Ciências Biológicas Universidade Federal de Ouro Preto, Minas Gerais, Brazil; 2 Departamento de Bioquímica e Biologia Molecular, Universidade Federal de Viçosa, Minas Gerais, Brazil; 3 Departamento de Física e Informática, Instituto de Física de São Carlos, Universidade de São Paulo, São Paulo, Brazil; 4 Departamento de Bioquímica, Instituto de Química, Universidade de São Paulo, São Paulo, Brazil; Universidade Federal do Rio de Janeiro, Brazil

## Abstract

**Background:**

The protozoan *Trypanosoma cruzi* is the causative agent of Chagas disease. There are no vaccines or effective treatment, especially in the chronic phase when most patients are diagnosed. There is a clear necessity to develop new drugs and strategies for the control and treatment of Chagas disease. Recent papers have suggested the ecto-nucleotidases (from CD39 family) from pathogenic agents as important virulence factors. In this study we evaluated the influence of Ecto-Nucleoside-Triphosphate-Diphosphohydrolase (Ecto-NTPDase) activity on infectivity and virulence of *T. cruzi* using both in vivo and in vitro models.

**Methodology/Principal Findings:**

We followed Ecto-NTPDase activities of Y strain infective forms (trypomastigotes) obtained during sequential sub-cultivation in mammalian cells. ATPase/ADPase activity ratios of cell-derived trypomastigotes decreased 3- to 6-fold and infectivity was substantially reduced during sequential sub-cultivation. Surprisingly, at third to fourth passages most of the cell-derived trypomastigotes could not penetrate mammalian cells and had differentiated into amastigote-like parasites that exhibited 3- to 4-fold lower levels of Ecto-NTPDase activities. To evidence the participation of *T. cruzi* Ecto-NTPDase1 in the infective process, we evaluated the effect of known Ecto-ATPDase inhibitors (ARL 67156, Gadolinium and Suramin), or anti-NTPDase-1 polyclonal antiserum on ATPase and ADPase hydrolytic activities in recombinant *T. cruzi* NTPDase-1 and in live trypomastigotes. All tests showed a partial inhibition of Ecto-ATPDase activities and a marked inhibition of trypomastigotes infectivity. Mice infections with Ecto-NTPDase-inhibited trypomastigotes produced lower levels of parasitemia and higher host survival than with non-inhibited control parasites.

**Conclusions/Significance:**

Our results suggest that Ecto-ATPDases act as facilitators of infection and virulence in vitro and in vivo and emerge as target candidates in chemotherapy of Chagas disease.

## Author Summary

The protozoan *Trypanosoma cruzi* is the causative agent of Chagas disease, an endemic zoonosis present in some countries of South and Central Americas. The World Health Organization estimates that 100 million people are at risk of acquiring this disease. The infection affects mainly muscle tissues in the heart and digestive tract. There are no vaccines or effective treatment, especially in the chronic phase when most patients are diagnosed, which makes a strong case for the development of new drugs to treat the disease. In this work we evaluate a family of proteins called Ecto-Nucleoside-Triphosphate-Diphosphohydrolase (Ecto-NTPDase) as new chemotherapy target to block *T. cruzi* infection in mammalian cells and in mice. We have used inhibitors and antibodies against this protein and demonstrated that *T. cruzi* Ecto-NTPDases act as facilitators of infection in mammalian cells and virulence factors in mice model. Two of the drugs used in this study (Suramin and Gadolinium) are currently used for other diseases in humans, supporting the possibility of their use in the treatment of Chagas disease.

## Introduction

*Trypanosoma cruzi* is the etiologic agent of Chagas disease, an endemic zoonosis present in some countries of South and Central Americas. WHO estimates suggested that 100 million people remain at risk of acquiring this infection [1]. There are no vaccines or effective treatment for this disease, especially in the chronic phase [2]. Many compounds are potential candidates to be used in the treatment for Chagas disease, such as TAK-187, D0870, albaconazole and allopurinol [2]. In spite of these, there is a clear necessity to develop new drugs and strategies for the control and treatment of Chagas disease [2]. From this point of view, virulence biomolecules, in particular those secreted or ecto-localized at the parasite's plasma membrane seem to be good targets. The concentrations of extra cellular nucleotides and their derivative molecules, such as adenosine and inosine are linked to ecto-nucleotidase activities of cells [3],[4]. The role of ecto-nucleotidases as the major biomolecules involved in the control of purinergic signaling were demonstrated in various models, such as the dominant role of CD39 in the modulation of inflammation and immune response in the Langerhans cells [5] and in cardioprotection and protective responses to hypoxia/ischemia in murine model [6],[7]. ATP has been previously demonstrated as a “danger” extracellular signal induced by pathogen infection or injury, and it is able to trigger different cellular events such as proliferation, differentiation and chemotaxis, release of cytokines or lysosomal constituents, and generation of reactive oxygen or nitrogen species [4]. Some authors believe that a high ecto-ATPase activity of pathogen is an adaptive parasitic behavior that made these organisms more virulent because they interfere with extracellular ATP signals [8]–[11].

Members of Ecto-NTPDase family are nucleotidases able to hydrolyze 5′-nucleoside tri- and/or diphosphates; the main role of these enzymes is the termination of purinergic signaling [12]. NTPDases are ubiquitous and were previously shown in other parasites including the trypanosomatides of genus *Leishmania* and in *T. brucei* [13]–[16]. Recent papers have suggested the ecto-nucleotidases from pathogenic agents, including parasites, and in a special way the Ecto-NTPDases (from CD39 family) as important virulence factors [9], [14], [17]–[21]. This hypothesis was clearly evidenced in *Toxoplasma gondii* and *Legionela pneumophila* [8],[11],[22]. In *T. gondii* the NTPase, a member of CD39 family, is produced as a soluble low activity tetrameric enzyme in the parasitophorous vacuole. Activation of this enzyme by dithiol agent (DTT) leads to depletion of host ATP levels and rapid exit of intracellular parasites from infected cells [8]. These data support the idea that activation of NTPase is an event related with the end of one intracellular parasite life cycle and the start of another cellular infection. In other words, NTPase activity would act as a timer and is crucial to *T. gondii* infection. In addition, Naakar and coworkers showed that lowering the expression of NTPDase by RNA antisense technology inhibits *T. gondii* proliferation in *in vitro* infection [22]. In *L. pneumophila* it was demonstrated that an Ecto-NTPDase, similar to CD39, is essential for intracellular bacterial multiplication. The authors showed that this pathogenic bacterium has two Ecto-NTPDases (lpg0971 and lpg1905), and only the product of lpg1905 is implicated with *in vitro* intracellular multiplication and mice virulence [11],[23].

*T. cruzi* is another example of pathogenic agent in which Ecto-NTPDase was suggested as a virulence factor. The first evidence of an ecto-nucleotidase at the *T. cruzi* surface was the demonstration of an ecto-ATPase activity in intact parasites that was partially sensitive to 4,4′-diisothiocyanatestilbene-2,2′-disulfonic acid (DIDs) [24]. Subsequently, three papers showed evidence that surface *T. cruzi* ecto-ATPase could modulate parasite-host interaction [10],[17],[25]. Bisaggio and co-workers [10] showed that treatment with ecto-ATPase inhibitors (Suramin and DIDs) lead to inhibition of ATPase activity, adhesion and internalization of parasites in macrophage *in vitro* infection [10]. In addition, the over-expression of ecto-ATPase activity was followed by a dramatic increase in parasite adhesion to resident macrophages.

In 2004 our group obtained the first biochemical characterization and immunolocalization of an Ecto-NTPDase on the *T. cruzi* surface [17]. In the same work we isolated a cDNA encoding an Ecto-NTPDase homologue to CD39 family enzymes. Because trypomastigotes presented higher levels of ecto-ATPase activity than epimastigotes and because the literature clearly pointed to ATP as a pro-inflammatory molecule we suggested that ecto-ATPase activity could be related with parasite virulence [17].

In the present work, we show that a high ratio of ecto-ATP/ADP hydrolysis is important to maintain the capacity of parasites to infect mammalian VERO cells. Additionally, Ecto-NTPDase inhibition modulates infectivity and virulence to mice, suggesting that NTPDase is a facilitator of *T. cruzi* infection.

## Methods

### Reagents

Giemsa was purchased from Merck (D-6100 Darmstadt, Germany). ARL67156, Suramin, GdCl3, ATP, ADP and AMP were purchased from Sigma Chemical Co. (St. Louis, MO). Distilled water was deionized using a MilliQ system (Millipore Corp., Bedford, MA) and was used in the preparation of all solutions.

### Parasites

Different *T. cruzi* strains, Y, CL, Be-62 (all from the *T. cruzi* II lineage) and CL Brener (a hybrid cloned strain) were maintained for successive blood passages in mice.

### Cell culture infections

Vero cells lines (carcinoma-derived African green monkey fibroblast cells) were seeded in 75 cm^2^ flasks at a density of 5×10^4^ and sustained in RPMI 1640 medium (GIBCO BRL) supplemented with 5% fetal calf serum (FCS, from CULTILAB, Campinas, SP, Brazil) and 1 mM L-glutamine (Sigma Aldrich). After 48 h of plating, the cultures were infected with bloodstream Y strain trypomastigotes harvested from *T. cruzi*-infected Swiss mice by orbital venous sinus puncture on the day of peak parasitemia employing a parasite/host cell ratio of 10∶1. After infection the cell cultures were maintained at 37°C in 5% CO_2_ atmosphere for 24 h for parasite internalization. After this time, cells were washed three times with phosphate buffered saline (PBS) solution, RPMI medium with 1% fetal calf serum (15 mL) was added and cells were incubated at 33°C, 5% CO_2_ atmosphere, for completion of the intracellular cell cycle of parasites. Vero cell-derived trypomastigotes were isolated from culture supernatants of infected cells after centrifugation at 2500×g for 15 min. The recently released cell-derived trypomastigotes were used for infection of new cells grown in 75 cm^2^ flasks employing a parasite/host cell ratio of 10∶1 and maintained for four sub-cultivation passages in Vero cells.

Parasites derived from cells infected with bloodstream trypomastigotes were called trypomastigotes of first cell passage (P1), and those obtained from the successive sub-inoculations performed with parasites derived from Vero cells were denominated trypomastigotes of 2^nd^, 3^rd^ and 4^th^ passages (P2, P3, P4).

CL, Be-62 or CL-Brener parasites were recovered only after the first passage (P1), Y strain parasites were recovered in P1 for “in vitro” infectivity assays and in vivo infectivity and virulence determination using Swiss mice as experimental model. Afterwards the Y strain was maintained until the fourth passage in the continuous cultivation assay.

### Ecto-NTPDase activity measurements

Intact live parasites were washed twice in 0.9% NaCl and suspended (1.0×10^8^ cells/ml) in nucleotidase reaction medium without nucleotides (116 mM NaCl, 5.4 mM KCl, 5.6 mM D-glucose, 50 mM Hepes-Tris buffer, pH 7.2). The assays were carried out in 125 µL total reaction volume. The reactions were started with the addition of 2.5 mM ATP or ADP, in the presence of 5 mM MgCl_2_ and were carried out as detailed elsewhere [14]. For the in vitro hydrolytic activity assays performed in the presence of specific ecto-ATPase inhibitors or polyclonal antiserum anti-NTPDase-1, parasites were exposed during the entire experimental time to the concentrations described in the Results. The ecto-nucleotidase activities were determined by measurement of inorganic phosphate (Pi) released to the medium after 1 hour of incubation at 37°C [26].

### Bacterial heterologous expression and purification of *T. cruzi* NTPDase-1

The recombinant *T. cruzi* apyrase NTPDase-1 (Accession No. AY540630) was expressed in bacterial heterologous system by transfer of the cDNA (1770 bp) encoding the predicted soluble portion of *T. cruzi* NTPDase-1 to the expression vector pET21b (Novagen). Cloning in the correct frame was confirmed by partial sequencing of recombinant plasmid, using T3 and T7 primers and BigDyeET-terminator kit in the MegaBace 500 apparatus, according to the manufacturer (GE Amersham Biosciences). The cloned sequence excluded the portion encoding the previously predicted putative amino-terminal signal peptide [17]. The recombinant plasmid pET21b-Tc-NTPDase-1 was used for transforming *Escherichia coli* BL21 (DE3). This vector adds a hexa-histidine sequence (Hexa-HIS) at the carboxyl-terminal portion of the resulting fusion protein, a tag that was used as target for purification of recombinant protein using nickel affinity chromatography Ni-NTA-agarose (GE-Amersham). Protein expression was induced with 1 mM IPTG during 1 hour at 37°C and 200 rpm. Purification and protein refolding were performed following previously described protocols [27].

### Anti-*T. cruzi*-NTPDase-1 polyclonal antiserum production

The purified recombinant protein (rNTPDase-1) was used for producing polyclonal antiserum by immunization of a female rabbit. Previous to the immunization a blood sample was obtained (3 mL) from the ear marginal vein (negative control). Purified recombinant NTPDase-1 (0.5 mg in 0.5 mL complete Freund's adjuvant- Sigma) was inoculated by intradermal route. After three weeks, another dose was inoculated (0.5 mg NTPDase-1 in 0.5 mL incomplete Freund's adjuvant-Sigma). The immune serum was recovered after an interval of 15 days. Blood was collected by puncture of the ear marginal vein and centrifuged at 3000 rpm for 10 min at room temperature; the serum supernatant was distributed in 1.5 ml aliquots. The pre-immune serum and immune antiserum were used in western blotting analysis and only immune serum was able to recognize the recombinant NTPDase-1 (data not shown). Both sera were treated for the complete inactivation of the complement system (CS) in order to be used in “in vitro” infectivity assays and NTPDase-1 inhibition assays. For this purpose, the pre-immune and immune sera were warmed to 56°C for 30 min. After CS inactivation these samples were stored in aliquots at −20°C.

### *T. cruzi* mammalian cell invasion assays (Infectivity assays)

*T. cruzi* Y strain [28] was maintained cyclically in mice as described above. VERO cells were grown at 37°C in RPMI 1640 medium (Sigma) supplemented with 5% fetal calf serum, garamycin (10 mg/ml) in a humidified 5% CO_2_ atmosphere. In vitro host cell invasion assays were carried out as detailed elsewhere [29], using first passage trypomastigotes (P1). Briefly, 5×10^5^ trypomastigotes from Y strain were placed in each well of 24-well plates containing 13-mm round glass cover slips coated with 5×10^4^ Vero cells (10∶1). After 24 h of infection, the cover slips were washed three times with 0.9% saline and stained with Giemsa (Merck). The numbers of infected cells and amastigotes per infected cell were counted in at least 300 cells, in quadruplicate. In experiments using trypomastigotes treated with NTPDase inhibitors (ARL67156, GdCl_3_, Suramin), parasites were recovered after one VERO cell passage, washed in sterilized saline solution and suspended in RPMI containing different concentrations of inhibitors. After 10 min of exposure to the inhibitor, parasites were recovered, suspended in new RPMI medium devoid of inhibitor and used for infecting VERO cells monolayer. The Vero cells were infected as described above. Samples of Y strain P1 trypomastigotes were produced using independent sets of mouse blood containing Y strain trypomastigotes.

### *T. cruzi in vivo* infection assay (Virulence assay)

All procedures and experimental animal protocols were conducted in accordance with the COBEA (Brazilian School of Animal Experimentation) and behavior instructions for the use of animals in research. For in vivo assays, groups of ten mice were inoculated with 5×10^3^ culture-derived P1-trypomastigotes of Y *T. cruzi* strain treated or not with ecto-nucleotidase inhibitors ARL67156 (300 µM), GdCl_3_ (300 µM) or Suramin (100 µM and 1000 µM). A second confirmatory assay was performed using the same concentrations of ARL67156 (300 µM), GdCl_3_ (300 µM) and only the most effective concentration of Suramin (1000 µM) using groups of six mice. Treatment with inhibitors was performed using 2×10^5^ parasites/ml during 10 min in MEM with 1% fetal calf serum and the respective ecto-nucleotidase inhibitors. After treatment parasites were recovered by centrifugation at 3500 rpm for 15 min, suspended in the same medium without inhibitors and used for infecting mice as described above. Parasitemia was evaluated by examination of fresh blood collected from the mouse-tail, starting from day 4^th^ post infection. The number of parasites was calculated as previously described [30]. Curves were plotted using the mean of the parasitemia obtained from six mice. Mortality rate was cumulative and expressed as a percentage of deaths within the period of 120 days after inoculation.

### Statistical analysis

Except when mentioned, all hydrolytic activity experiments were performed in triplicate, with similar results obtained in at least three separate cell suspensions. Statistical significance was determined by Student's t test. Differences were considered significant at p<0.05. Data were expressed as average±standard deviation.

## Results

### Ecto-NTPDase activities are diverse in distinct strains of *T. cruzi*

**Figure 1:**
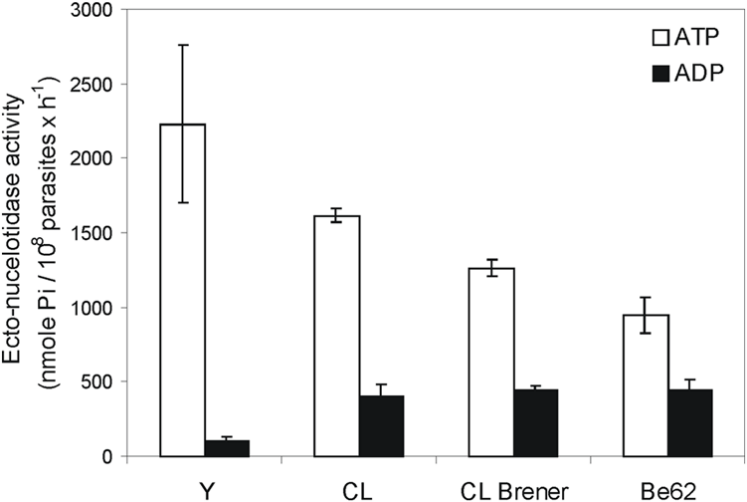
Ecto-nucleotidase activities of trypomastigotes from different strains/clone of *T. cruzi*. Trypomastigotes were obtained from the first VERO cells passage (P1). The Ecto-ATPDase activities were measured at 37°C during 1 hour. Data are mean±SE of two independent experiments in triplicate.

Blood trypomastigotes from Y, Be-62 and CL strains and the clone CL-Brener were used for infecting VERO cell cultures. Parasites obtained after one passage (P1) were used for measuring Ecto-ATPDase hydrolytic activity. The different strains exhibited distinct levels of ATP and ADP hydrolysis (Figure 1). Y strain presented the highest levels of ATP hydrolysis and lowest ADP hydrolysis and was chosen for further *in vitro* and *in vivo* infection assays.

### Y strain Ecto-NTPDase activity and infectivity decreases during continuous VERO cells cultivation

**Figure 2:**
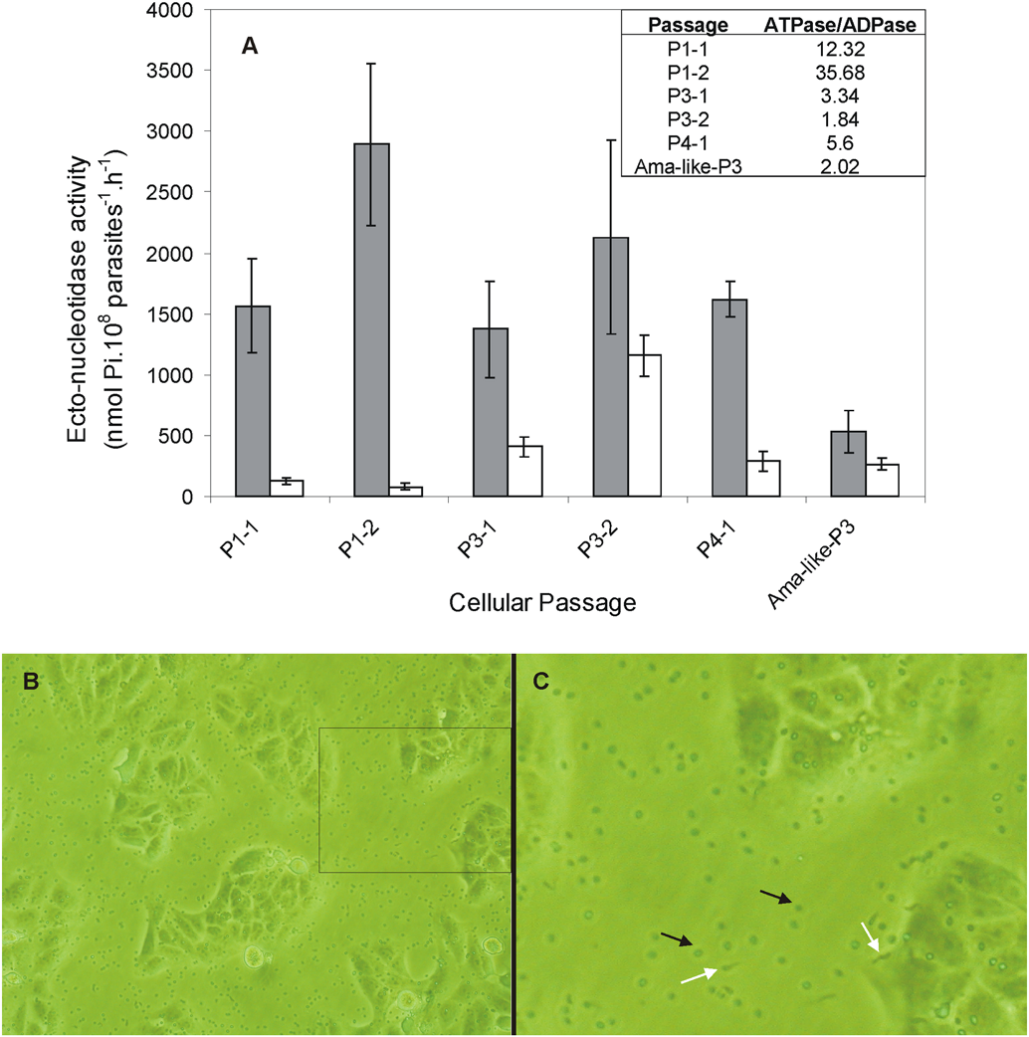
*T. cruzi* infectivity and ecto-ATPase/ADPase ratio decrease during in vitro cultivation. A) Ecto-ATPDase (solid bars) and Ecto-ADPDase (open bars) activities from live trypomastigotes from different cellular passages (P1, P3 and P4). P1-1 and P1-2 are the first and second massive exits of parasites from the 1^st^ passage; in the same way P3-1 and P3-2 are the first and second massive exits of parasites for the 3^rd^ passage. Data are mean±SE of triplicate assays from one experiment. The inset shows the ATPase/ADPase hydrolytic activities ratio. B) Microphotograph of infected VERO cells culture after 24 h of parasite-cell interaction at the 3^rd^ to 4^th^ passage. Spherical bodies are non-internalized amastigote-like parasites. C) Zoom from box section shown in B. Black arrow exemplifies a non-internalized amastigote-like and white arrow a non-internalized trypomastigote parasite.

In order to establish a possible correlation with ecto-nucleotidase activity and infectivity capacity, we started a continuous *in vitro* cultivation of Y trypomastigotes in VERO cell cultures and analyzed Ecto-ATPDase activity. Each global exit of parasites to the culture supernatant after completion of the intracellular life cycle was named as “one passage (P)”. Because Y is a polyclonal strain we observed two days of massive parasite exit from VERO cells; samples for each day of cell exit for the 1^st^ and 3^rd^ passages were collected and called PN-X, were N indicates the passage and X indicates the day from the beginning of cell exit in each passage. We detected a marked 3- to 6-fold decrease in the ATPase/ADPase ratio (Figure 2A, inset) and in the number of parasites that infected cells at the 3^rd^ to 4^th^ passage when compared to the 1^st^ passage parasites (Figures 2B, 2C). Because of this observed low infection from 3^rd^ to 4^th^ passage, we were unable to recover sufficient parasites to perform enzymatic assays for the 5^th^ and later passages. Curiously, ecto-ATPase activity ranged from 1400 to 2800 nmol Pi.10^8^ parasites^−1^.h^−1^ (P1, P3, P4) and it did not decrease significantly in trypomastigotes that could complete infection and intra cellular cycle in all analyzed passages (Figure 2A). On the other hand, ecto-ADPase activity increased from 50–100 nmol Pi.10^8^ parasites^−1^.h^−1^ in the first passage to 300–1200 nmol Pi.10^8^ parasites^−1^.h^−1^ in passages 3 and 4 (Figure 2A), reflecting in a significantly decreased ecto-ATP/ADPase ratio (Figure 2A, inset).

We did not measure infectivity directly during passages cultivation, but it could be clearly observed that the number of parasites from the 3^rd^ to 4^th^ passage penetrating VERO cells was very low. The majority of parasites did not infect cells and differentiated to amastigote-like in the culture supernatant (Figure 2B, 2C). We recovered these parasites and observed that the population was comprised of about 80% amastigote-like and 20% trypomastigotes that could not penetrate VERO cells. We compared the ecto-nucleotidase activity of these parasites (indicated as Ama-like-P3 on Figure 2A) with that of infective P4 trypomastigotes (parasites that penetrated cells, concluded the cellular life cycle and were recovered from VERO cell culture supernatant). In Figure 2A (inset) we show that these non-penetrating amastigote-like parasites have an ecto-ATPase/ADPase ratio of 2.0, which is 2.5-fold lower than infective P4-trypomastigotes (ratio = 5.6). Overall, these results suggested that a high ATPase/ADPase ratio (in the range of 12 to 36) seems to be a requirement for internalization of parasites. We speculate that the absence of mammalian host factors in the culture medium could influence the differentiation process, generating non-infective amastigote-like parasites with reduced Ecto-ATPDase activity.

### Known ecto-ATPDase inhibitors decrease *T. cruzi* Ecto-ATPDase activities and *in vitro* infectivity

In face of the above results, we decided to investigate the effect of known ecto-ATDPase inhibitors on *T. cruzi* infectivity and hydrolytic activity. We tested the action of Ecto-ATPDase inhibitors directly on live P1-trypomastigotes from Y strain. The inhibitors tested were: ARL67156 (6-N,N-Diethyl-β-γ-dibromomethylene-D-adenosine-5-triphosphate) considered to be a selective inhibitor of ecto-ATPase [31]; Gadolinium, a lanthanide related with extra- and intracellular ATP action and able to inhibit Ecto-NTPDase from *Torpedo* electric organ [32] and Suramin, a polysulfonated naphthylurea compound that was previously demonstrated to inhibit *T. cruzi* Ecto-ATPDase [10].

Intact parasites were incubated for 10 min with different concentrations of the above inhibitors (0, 100, 300, 500 µM) and were subsequently recovered by centrifugation and suspended in fresh buffer free of inhibitors. These pre-treated parasites were used for *in vivo* Ecto-ATPDase hydrolysis assays and in experimental VERO cells infection.

**Figure 3:**
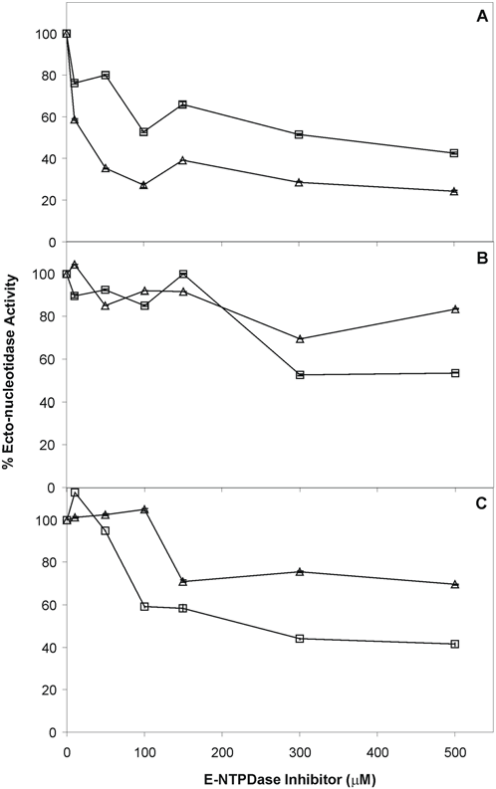
Effect of inhibitors in the ecto-ATPDase activity of Y strain P1 trypomastigotes. Parasites were pre-incubated for 10 min with different concentrations of Suramin (Panel A), ARL 67156 (Panel B) or Gadolinium (Panel C) and ecto-ATPDase activities were measured. Ecto-ATPase (Δ) and ecto-ADPase (□) activities are expressed as percentage of control activity (without inhibitors). Data are mean±standard error of two independent experiments in triplicate.

We observed that all drugs tested were able to partially inhibit ATPase and ADPase activities. Higher *in vivo* inhibition of *T. cruzi* ecto-ATPase (approximately 75%) was achieved with Suramin 100 µM (Figure 3A). Gadolinium and ARL67156 inhibited only approximately 30% of this activity at 300 and 500 µM respectively (Figure 3B and 3C). On the other hand ADPase activity was more effectively inhibited (60%) by Suramin and Gadolinium at 500 µM; ARL 67156 inhibited about 50% of ADPase activity at 300 µM (Figure 3A, 3B and 3C).

Infectivity of parasites treated with ATPDase inhibitors (300 µM ARL 67156, 300 µM Gadolinium or 100 µM Suramin) were evaluated in VERO cells. P1 trypomastigotes (Y strain) were pre-treated during 10 min with the indicated inhibitor concentrations. Parasites were recovered by centrifugation, washed in appropriate medium and used for infecting VERO cell monolayers. After 24 hours of interaction (parasites-cells) the non-internalized parasites were discarded. The slides were covered with new growth medium and incubated during an additional 24 h. After staining, levels of cell infection and the number of intracellular amastigotes per infected cells were measured.

**Figure 4:**
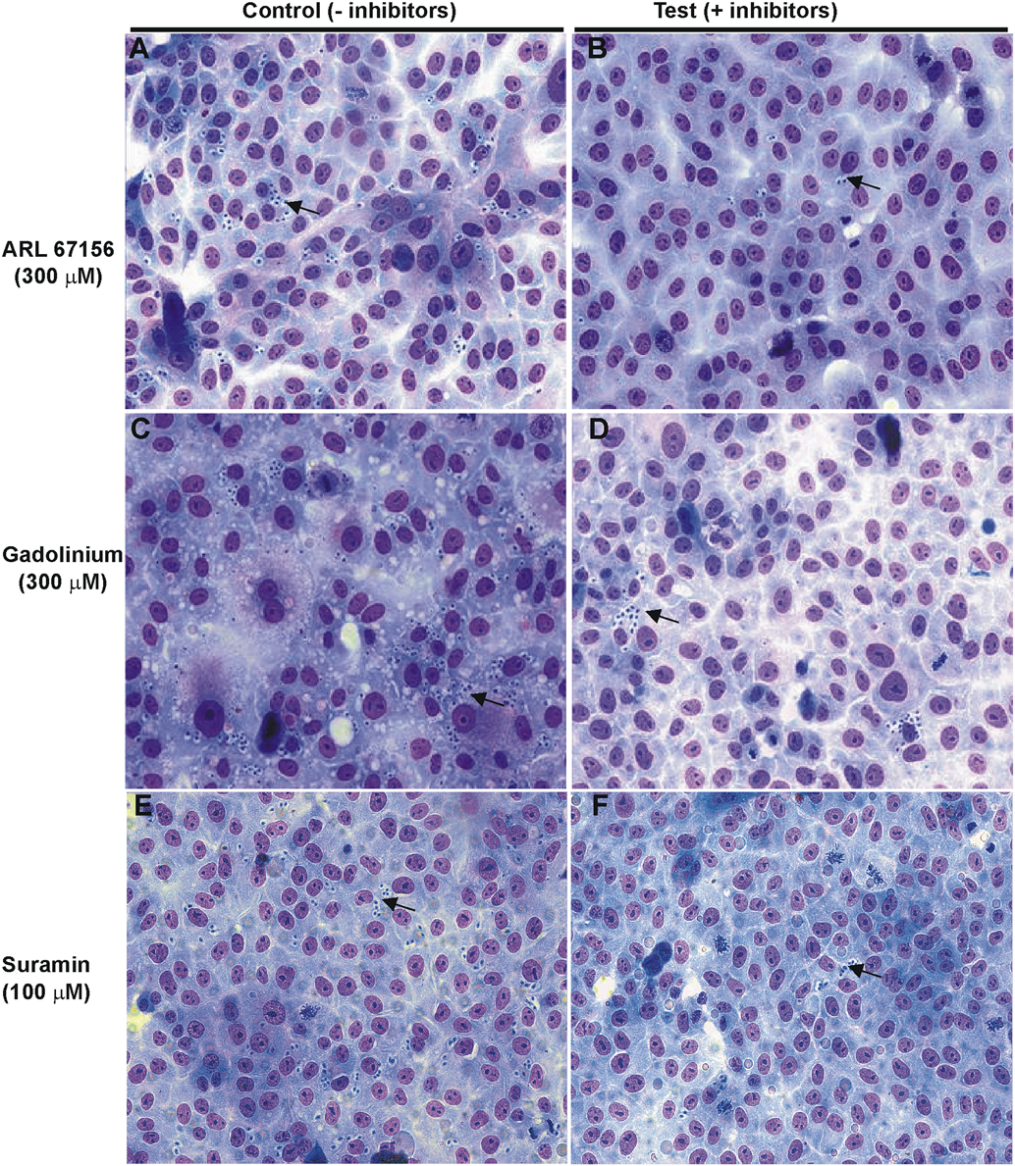
Effects of ecto-ATPDase inhibitors on *in vitro T. cruzi* infectivity. Microphotographs of VERO cell monolayers infected with trypomastigotes not treated (panels A,C,E) or treated with Ecto-ATPDase inhibitors (panels B,D,F). P1 trypomastigotes (Y strain) were pre-treated with ARL 67156, Gadolinium or Suramin at the indicated concentrations, resuspended in medium without inhibitor and used in VERO cells invasion assays. After 24 hours post infection each assay slide was washed and stained with Giemsa. The arrows indicate many internalized amastigotes.

Suramin was the most effective *in vitro* infectivity inhibitor, as revealed by a significant 71% reduction in the number of infected cells per 300 cells (Table 1), which is also visually documented in microphotographs of VERO cells exposed to control or Suramin pre-treated parasites (Figures 4E and 4F, respectively). It should be noted that pre-treatment of parasites with Suramin caused an infectivity blockage while not affecting the intracellular survival of those parasites that managed to penetrate the cells, as evidenced by a similar number of parasites per infected cell detected in the experiment (Table 1). ARL 67156 and Gadolinium caused 42% and 65% infectivity inhibition, respectively (Table 1, Figure 4A, 4B, 4C, 4D). The results show that only Suramin and Gadolinium lead to significant decrements in *T. cruzi* infectivity. These observed effects resulted from a direct action of drugs on the parasite rather than derived from a response of VERO cells to the drugs, since the cells did not have direct contact with the drugs. Similar to Suramin, ARL 67156 pre-treatment did not affect the detected number of parasites per infected cell, whereas GdCl_3_ pre-treatment caused a significant decrement in the number of parasites per infected cell (Table 1). This latter observation could be related with the action of GdCl_3_ inhibitor upon target biomolecules involved with both infection and differentiation processes.

**Table 1:** Effect of apyrase inhibitors on *in vitro* infectivity of trypomastigotes.

Ecto-NTPDase Inhibitor	# Parasites/infected cell		# Infected cells/300 cells		Infectivity Inhibition (%)
	−	+	−	+	
ARL 67156 (300 µM)	2.95±0.32	2.35±0.39	28.92±5.50	17.54±3.52	**42±1.17**
Gadolinium (300 µM)	4.65±0.38	3.42±0.23*	106.14±5.66	36.72±8.71	65±2.90*
Suramin (100 µM)	3.20±0.52	3.24±0.24	64.93±3.65	13.18±2.74	71±0.85*

Percent inhibition of infection was calculated for each inhibitor assay relative to control data. Data reflect the mean±SE from three analyzed slides. Asterisks indicate significant differences (p<0.05) between the control without inhibitor and the test with ATPDase inhibitor.

### *T. cruzi* Ecto-NTPDase-1 is inhibited by Suramin

**Figure 5:**
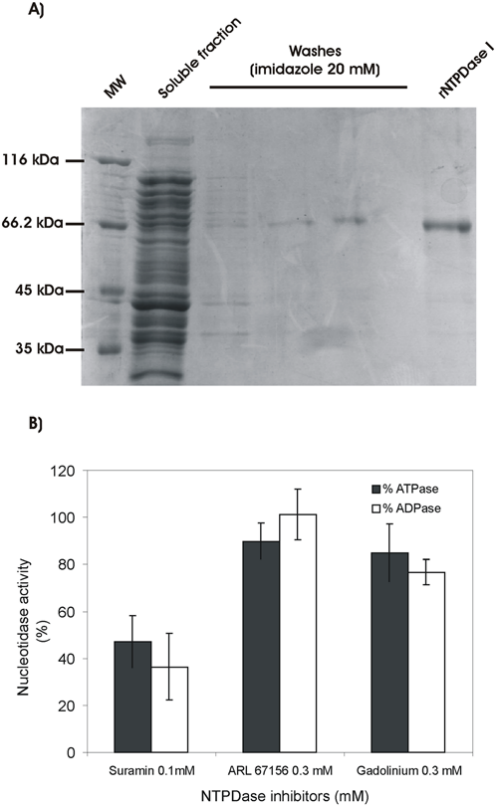
Heterologous expression of *T. cruzi* Ecto-NTPDase-1 and the effect of ecto-ATPase inhibitors on purified protein. A) SDS-PAGE stained with coomassie blue. A 15 µL sample of each step of production and purification of Ecto-NTPDase-1 was applied in each lane, showing the purified protein with approx. 66 kDa. MW, molecular weight markers. B) ATPDase activity of purified Ecto-NTPDase-1 in the presence of ecto-ATPase inhibitors. The results represent the percent activity with inhibitor related to the activities in the absence of inhibitors. Data are mean±SE of two independent experiments, each assayed in triplicate.

We have only identified one *T. cruzi* NTP-diphosphohydrolase in the parasite genome, named NTPDase-1 [17]. In light of the results described in the previous sections, we decided to study if NTPDase-1 could be responsible for the Ecto-ATPDase activity correlated with parasite infectivity. We obtained the recombinant *T. cruzi* NTPDase-1 (∼66 kDa) by heterologous expression, purified it by affinity chromatography (Figure 5A) and evaluated its ATPDase activity in presence or absence of the previously described Ecto-NTPDase inhibitors. The specific ATPase and ADPase activities were 24.0±8.4 and 14.3±6.9 nmol Pi. h^−1^ .µg Prot^−1^, or 0.4 and 0.24 µmol Pi.min^−1^.mgProt^−1^. Specific activities are in the range of recombinant human enzymes [33]. Concerning only the average activity values the ATPase/ADPase ratio is 1.7, similar to mouse (1.9) and human (1.9) NTPDase1 isoforms [33]. We show in Figure 5B that only Suramin caused significant levels of nucleotidase inhibition of this enzyme. The level of Suramin inhibition on recombinant purified NTPDase-1 (40 to 50%) was very similar to the extent of Suramin inhibition of hydrolytic activity on live parasites (Figure 3), suggesting that Suramin blockage of trypomastigote infectivity (Table 1) could be related with its effect on *T. cruzi* NTPDase-1. On the other hand ARL 67156 and Gadolinium showed no effect on the activity of this recombinant enzyme, suggesting that other enzymes with NTPDase activity sensitive to these inhibitors may exist at the *T. cruzi* surface.

### Polyclonal antiserum produced against NTPDase-1 inhibited *in vitro T. cruzi* infection

We produced a polyclonal antiserum anti- *T. cruzi* NTPDase-1 using the purified recombinant protein as antigen for immunization of rabbits. This serum gives a strong signal in immuno-fluorescence experiments; in contrast no signal is obtained from the negative control pre-immune serum, thus clearly indicating that immunization of rabbits with the antigen has produced antibodies against NTPDase-1 (Cunha et al., manuscript in preparation).

*In vitro* assays using the antiserum fail to produce any inhibitory action towards the recombinant protein enzymatic activity (data not shown). This indicates that although the produced antibodies are able to bind to NTPDase-1, they must target regions that are not essential for enzymatic activity.

A complement system (CS) inhibition pre-treatment of the pre-immune and immune sera was performed as described in material and methods, before its use in infectivity assays. Y strain P1 trypomastigotes were submitted to pre-incubation with CS inactivated antisera, and parasites were used for infecting VERO cell monolayers. We observed that the antiserum assayed at 1∶50 and 1∶100 significantly inhibited the infectivity of parasites (Table 2).

**Table 2:** Effect of antiserum anti-NTPDase-1-HexaHIS or pre-immune serum on *in vitro* infectivity of trypomastigotes.

	Antiserum anti-NTPDase-1-HexaHIS				Pre-immune serum			
	1∶50		1∶100		1∶50		1∶100	
	−	+	−	+	−	+	−	+
**# Parasites/infected cell**	2.11±0.31	2.04±0.50	1.47±0.30	2.20±0.13	5.88±0.82	6.40±1.30	5.88±0.82	5.34±0.76
**# Infected cells/300 cells**	18.9±3.22	8.83±1.75	17.4±3.53	8.96±1.16	109.0±6.62	135.0±17.1	109.0±6.62	122.1±11.5
**Infected cells inhibition (%)**	**53±0.58** *		**49±0.38** *		**0**		**0**	

*: Percent inhibition of infection was calculated for each inhibition assay relative to control data. Data reflect the mean±SE from three analyzed slides. Asterisks indicate significant differences (p<0.05) between the control without antiserum (−) and the tests with antiserum (+).

### Ecto-NTPDase inhibitors decrease *T. cruzi* mouse infection and virulence

**Figure 6:**
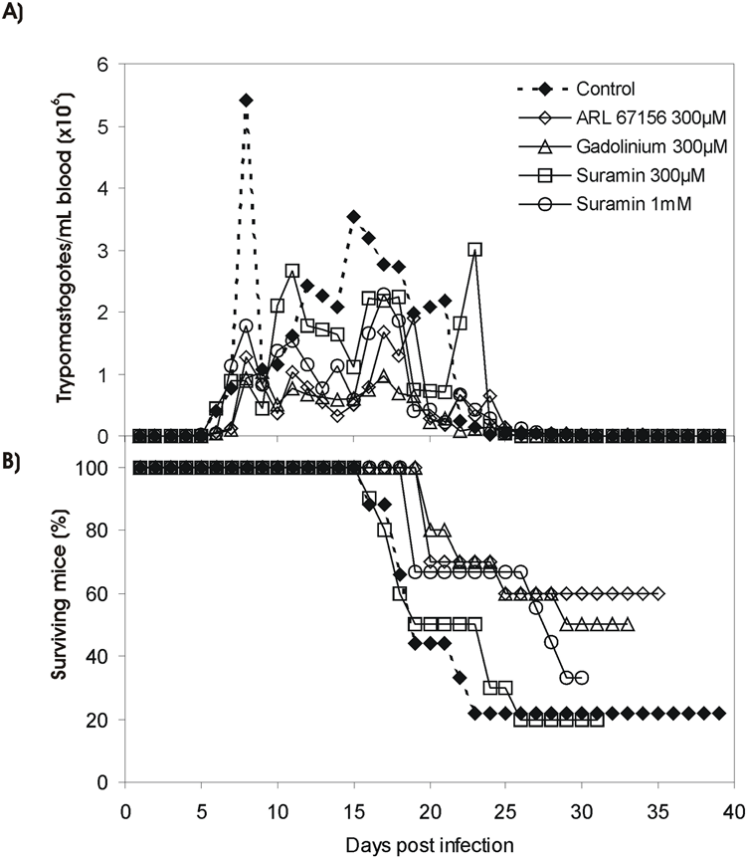
Inhibition of *T. cruzi* Ecto-ATPDases decreased virulence to mouse. A) parasitemia curves and B) mortality in Swiss mice infected with 5,000 parasites/0.1 mL blood (Y strain P1 trypomastigotes). Parasites were pre-treated with ARL67156, Gadolinium or Suramin as indicated; a negative control assay is included, omitting the parasites' drug pre-treatment. Data are from one experiment using the mean value of a group of 10 mice in each treatment.

To confirm the results from experiments using VERO cells as model, we performed experimental *in vivo* infection assays in mice using P1 Y trypomastigotes pre-treated with the optimal concentration of Ecto-NTPDase inhibitors that blocked in vitro infection (Table 1 and Figure 4). A parallel negative control experiment was performed by omitting drug pre-treatment of parasites. All data are mostly related with the action of drugs directly on parasites rather than on mice, because parasites are pre-treated with inhibitors, washed and finally inoculated in the animals. Figures 6A and B show the parasitemia and mortality verified in the tested animals, respectively. Data clearly shows that pre-exposure of parasites to ARL 67156 (300 µM) or Gadolinium (300 µM) resulted in a decreased parasitemia and increased host animal's survival. The most effective drug was ARL 67156, which resulted in about 60% mice survival when compared with mice infected with non-treated parasites (control). Pre-treatment of parasites with Suramin at 100 µM (data not shown) and 300 µM (Figure 6B) did not result in significant protection, while pre-exposure of parasites to 1 mM Suramin resulted in a significant decrease in parasitemia as well as a prolonged time of survival of infected mice (Figure 6A, 6B). A second confirmatory experiment was performed and gave similar results (data not shown).

## Discussion

The present work shows evidence that continuing cultivation of trypomastigotes in VERO cells caused an infectivity decrease during cultivation and that only a small fraction of parasites was able to infect VERO cells and conclude another cellular cycle at the third to fourth passage. Parasites (trypomastigotes) that completed this passage presented 2.5-fold higher levels of ecto-ATPase activities when compared with amastigote-like parasites that could not penetrate into VERO cells. These data reinforce that a high ecto-ATPase activity is important for infectivity and suggest that the absence of host factors leads to loss of infectivity factors from *T. cruzi*. Similar suggestion was made by Bêrredo-Pinho et al. [14] when the ecto-ATPase activity was assayed on promastigotes from *Leishmania amazonensis* and showed that higher passages in acellular medium lead to low ecto-ATPase levels and avirulence [14]. However, as we have used a single strain (Y) in our experiments, verification of correlation between infectivity and Ecto-ATPDase activity in other strains may provide further support to our hypothesis.

In order to evaluate the importance of Ecto-NTPDase activity in *T. cruzi* infectivity we performed experiments with three known enzyme inhibitors. Analyzing Ecto-ATPDase inhibitory effects of these compounds we observed that all were able to partially inhibit both ecto-ATPase and -ADPase on live parasites, but the effect of ARL67156 and Gadolinium on the ecto-ATPase was lower than that observed for Suramin. In parallel, we observed that P1 trypomastigotes treated with ecto-nucleotidase inhibitors showed significant decrements of *in vitro* infectivity (Table 1) and *in vivo* virulence (Figure 6), suggesting the importance of Ecto-ATPDase activity in these processes. We cannot exclude the possibility that inhibitors used in this work had an additional action on other molecular targets. It has been shown that P2 receptors are susceptible to blockage by Suramin [34], but even if that is the case our data would still point out the importance of the extracellular nucleotide metabolism for *T. cruzi*, as demonstrated for other parasites [35]. Further studies including the use of more selective ENTPDase inhibititors, such as Polyoxometalates [36] and anthraquinone derivatives from bromaminic acid [37], are still necessary to determine the exact molecular mechanism inducing the loss of infectivity and virulence observed in our experiments.

Parasites have been incubated with high concentrations of inhibitor, but the *in vitro* experiments were performed in drug-free medium after the centrifugation of parasites. The persistence of effects from drugs on the parasite even after their removal suggests that the drugs might have triggered persistent changes in the parasite's metabolism that had a very slow recovery rate.

Considering the important roles of ATP, ADP and Adenosine as extracellular molecules in modulating the inflammatory process and platelet aggregation [5], [38]–[40] and the proposed role of NTPDases in host pathogen interactions [41], it is possible to speculate that some of the *in vivo* effects observed might be correlated to the impairment of the parasites' ability to modulate the levels of these nucleotides by inhibition of NTPDase. Further experiments are warranted to substantiate this hypothesis.

The present work shows the first biochemical demonstration of recombinant *T. cruzi* Ecto-NTPDase-1 activity. Enzymatic inhibition of this recombinant protein in the presence of Suramin, suggests that the effects of this drug on intact parasites, namely blockage of cell infection may be due to targeting of this inhibitor to the Ecto-NTPDase-1 on their surface. Polyclonal antiserum to *T.cruzi* Ecto-NTPDase also blocked infection of the cells. It should be noted that the antibodies did not display any direct inhibitory effect on enzymatic activity of the recombinant NTPDase-1, under controlled *in vitro* conditions. Considering that during the infection process the parasite will be exposed to diverse environmental conditions, it is possible that even though the antibody was not directly targeted to the NTPDase-1 active site, it might bind to modulatory regions preventing the response of the enzyme to certain stimuli. Alternatively, antibody binding might generate a steric hindrance at the parasite surface that prevents its adhesion to host cells.

Structural studies of NTPDase-1 and its use for the rational design of inhibitors could be a relevant strategy for the development of new drugs to treat the disease. In addition, the observed effect of antibodies on the parasites suggests that an effective immune response from the host could be mounted based on vaccination using this antigen. Further immunization experiments using this protein are necessary to verify its potential as a protective antigen.

In contrast, the observed ARL 67156 and Gadolinium inhibition of hydrolytic activities on live parasites could be related with another ecto-nucleotidase target, since they caused no inhibition of purified *T. cruzi* Ecto-NTPDase-1; this observation suggests the existence of other enzymes with Ecto-NTPDase activity at the parasite surface. We exhaustively searched for another apyrase/CD39 gene in the *T. cruzi* genome public database, including the *in silico* screening of 67 Mb of partial genome assembly [42] without any success. This indicates that targets for ARL 67156 and Gadolinium are either Ecto-NTPDases encoded by genes that are located in a portion of the genome not yet sequenced or are proteins with no detectable primary sequence similarity with Ecto-NTPDase. Alternatively, it is possible that Ecto-NTPDase-1 is in fact inhibited by these other compounds, but the recombinant version of this protein is insensitive due to alterations in its structure compared to the native form.

This paper shows the correlation of Ecto-ATPDase activity with *T. cruzi* infectivity and virulence. In addition, inhibitors of such activity tested here appear to interfere with the parasite infection process and emerge as possible new clinical drugs to Chagas disease treatment. Because Suramin and Gadolinium are currently used in unrelated human chemotherapy these drugs have already been tried for safety and may provide prompt options for development and use in Chagas disease treatment. Suramin is a current drug used in the treatment of Human African trypanosomiasis [43] and exhibits toxic effects to *T. cruzi* [44],[45]. Gadolinium is used as a contrast drug in magnetic resonance clinical imaging exams. (Gd)-based paramagnetic contrast agents are relatively safe when used in clinically recommended doses, however there is literature linking Gd-based paramagnetic contrast agents with nephrogenic systemic fibrosis (NSF) in patients with renal failure [46]. We believe that chemotherapy studies with these compounds and their association with the currently used drug Benznidazole in lower doses is warranted and may represent an alternative to treat Chagas patients.
